# Evaluation of the accuracy of serum MMP-9 as a test for colorectal cancer in a primary care population

**DOI:** 10.1186/1471-2407-6-258

**Published:** 2006-10-31

**Authors:** Sue Wilson, Michael JO Wakelam, Richard FD Hobbs, Angela V Ryan, Janet A Dunn, Val D Redman, Fiona Patrick, Lynne Colbourne, Ashley Martin, Tariq Ismail

**Affiliations:** 1 Department of Primary Care and General Practice, The University of Birmingham, Edgbaston, Birmingham, B15 2TT, UK; 2 University Hospital Birmingham Foundation NHS Trust, Queen Elizabeth Hospital, Birmingham, B15 2TH, UK; 3 Cancer Research UK Institute for Cancer Studies, The University of Birmingham, Edgbaston, Birmingham, B15 2TT, UK; 4 Clinical Trials Unit, University of Warwick, Health Sciences Research Institute, Medical School Building, Gibbett Hill Campus, Coventry, CV4 7AL, UK

## Abstract

**Background:**

Bowel cancer is common and is a major cause of death. Meta-analysis of randomised controlled trials estimates that screening for colorectal cancer using faecal occult blood (FOB) test reduces mortality from colorectal cancer by 16%. However, FOB testing has a low positive predictive value, with associated unnecessary cost, risk and anxiety from subsequent investigation, and is unacceptable to a proportion of the target population. Increased levels of an enzyme called matrix metalloproteinase 9 (MMP-9) have been found to be associated with colorectal cancer, and this can be measured from a blood sample. Serum MMP-9 is potentially an accurate, low risk and cost-effective population screening tool. This study aims to evaluate the accuracy of serum MMP-9 as a test for colorectal cancer in a primary care population.

**Methods/Design:**

People aged 50 to 69 years, who registered in participating general practices in the West Midlands Region, will be asked to complete a questionnaire that asks about symptoms. Respondents who describe any colorectal symptoms (except only abdominal bloating and/or anal symptoms) and are prepared to provide a blood sample for MMP9 estimation and undergo a colonoscopy (current gold standard investigation) will be recruited at GP based clinics by a research nurse. Those unfit for colonoscopy will be excluded. Colonoscopies will be undertaken in dedicated research clinics. The accuracy of MMP-9 will be assessed by comparing the MMP-9 level with the colonoscopy findings, and the combination of factors (e.g. symptoms and MMP-9 level) that best predict a diagnosis of malignancy (invasive disease or polyps) will be determined.

**Discussion:**

Colorectal cancer is a major cause of morbidity and mortality. Most colorectal cancers arise from adenomas and there is a period for early detection by screening, but available tests have risks, are unacceptable to many, have high false positive rates or are expensive.

This study will establish the potential of serum MMP-9 as a screening test for colorectal cancer. If it is confirmed as accurate and acceptable, this serum marker has the potential to assist with reducing the morbidity and mortality from colorectal cancer.

## Background

Colorectal cancer is a major public heath problem: it is the third and second most common invasive malignancy in men and women, respectively, in England [1]. There are over 28000 incident cases in England and over 14000 deaths in England and Wales each year [1,2].

The five year age-standardised relative survival rate is less than 50% for men and women with cancer of the colon [3]. Survival is strongly associated with stage at presentation. The five-year relative survival rate for people with localised cancer is 90%, whereas the rate for those with distant disease is only 9% [4]. Most colorectal cancers arise from adenomas, and estimates of the progression rate suggest that there is a long window period for early detection by screening and removal of the adenoma [5]. Colorectal cancer, therefore, meets key Wilson and Jungner criteria for a condition that may be a suitable target for screening [6].

Meta-analysis of randomised controlled trials estimates that screening for colorectal cancer using faecal occult blood (FOB) test reduces mortality from colorectal cancer by 16% [7], but a low positive predictive value (12% for colorectal cancer) [8] means that most positive tests are false with associated unnecessary cost, risk and anxiety from subsequent investigation. Although a UK study is underway to assess whether flexible sigmoidoscopy screening can reduce the incidence and mortality of colorectal cancer, it will be several years before results show if incidence/mortality are reduced [9]. Early findings are promising, but only part of the colon is screened, the test is costly and invasive, and it has low acceptability: less than 50% of people aged 50–75 years accepted an invitation for screening [10]. Colonoscopy is the most accurate way to detect pathology, but it requires secondary care resources, is unacceptable to some, and has an element of risk. Combined results from several studies suggest that the incidence of perforation and haemorrhage in a diagnostic colonoscopy are 1 in 603 and 1 in 1352, respectively [11]. A study in the USA found a perforation rate of 1 in 510 within seven days of colonoscopy in people aged 65 years or older, although the rate was lower (1 in 747) where no indication, for example diverticulosis, was identified [12]. The cost, low acceptability and risk make colonoscopy unsuitable as a routine screening test.

FOB testing also has relatively low acceptability, with reported uptake rates in the UK of only 60% [13]. A blood test is likely to be a more acceptable routine screening test than FOB, flexible sigmoidoscopy or colonoscopy. Matrix metalloproteinases (MMPs) are proteolytic enzymes that are associated with tissue remodelling in normal and pathological processes [14]. Over-expression of MMPs has been correlated with progression in many tumour types, and over-expression of MMP-9 has been found in colorectal adenomas [15] and carcinomas [16,17]. A significant positive correlation has also been found between MMP-9 and the stage of colorectal tumours [18]. MMP-9 may be detected by ELISA assay.

### Pilot work

We have completed a pilot study of 300 patients (all "urgent" referrals) attending a specialist colorectal clinic. In this high prevalence group (27% with pathology), the accuracy of serum MMP-9 for colorectal neoplasia was 73% (sensitivity 99%, specificity 63%, positive predictive value 50%, negative predictive value 99%). Serum MMP-9, therefore, appears to have the potential to be a useful screening test for colorectal neoplasia. There is a strong possibility, however, that our pilot was affected by spectrum bias. Although the sensitivity and specificity of a test are unaffected by disease prevalence, the performance of a diagnostic test can vary according to the spectrum of the population being tested, that is the severity and clinical presentation of the disease [19,20]. Evaluation of the accuracy and acceptability of serum MMP-9 in a primary care population, the target population for a potential screening test, is required to inform the utility of serum MMP-9 screening in the general population.

*Feasibility work *for this study has also been undertaken. One hundred and ninety eight questionnaires were distributed in December 2003. There was a return rate of 74% (n = 146), and 59% (n = 116) returned a completed questionnaire. Twenty four respondents (21% of 116) had symptoms other than only abdominal bloating and/or anal symptoms, and 13 of these people (54% of 24) agreed to take part in the study. Eleven, of the 13 agreeing to take part, were invited to a general practice research clinic, eight returned the availability slip and seven agreed to attend. Six of these people attended a research clinic and all were eligible and consented to have a blood sample and colonoscopy.

Our pilot work suggests that serum MMP-9 is acceptable and has a high sensitivity and negative predictive value. Colorectal cancer is a suitable candidate for mass screening and a bowel cancer screening programme will be introduced in the UK with national coverage planned by 2010 [21,22]. Currently available screening tests (FOB, flexible sigmoidoscopy, colonoscopy) are, however, not particularly acceptable to the general population and are either risky or have high false positive rates. Flexible sigmoidoscopy or colonoscopy screening would also be expensive and place a considerable burden on existing resources. Serum MMP-9 is potentially an accurate, low risk and cost-effective population screening tool. It is, therefore, important that we evaluate its usefulness in a primary care population.

### Aims and objectives

#### Aim

To evaluate the accuracy of serum MMP-9 as a test for colorectal cancer in a primary care population.

#### Objective

Primary objectives are to evaluate:

(1) the accuracy of serum MMP-9 as a test for colorectal cancer by comparison with colonoscopy.

(2) the acceptability of serum MMP-9 as a screening test for colorectal cancer in a primary care population.

Secondary objectives are:

(1) to describe the prevalence of lower gastrointestinal symptoms in a primary care population.

(2) to assess the appropriateness of current referral guidelines.

## Methods/Design

### Study design

Community based survey with embedded diagnostic test accuracy evaluation. Accuracy of MMP9 will be assessed by comparison with the gold standard, colonoscopy. A cohort aged 50–69 years with lower gastrointestinal symptoms will be identified using a questionnaire. People who are willing to participate will be given further information at a general practice based research clinic and informed consent will be obtained. Participants will have a blood sample for serum MMP-9 determination and a colonoscopy.

### Recruitment of practices

Up to 30 general practices will be recruited. Practices will be eligible to participate if they are in the catchment area of University Hospital Birmingham NHS Foundation Trust.

### Eligibility criteria

People aged 50–69 years registered with participating general practices.

### Exclusion criteria

The GP will exclude people who are under investigation or treatment for colorectal cancer, unfit for colonoscopy, unable to give informed consent, or who should not be invited to take part, in the GPs opinion, for any other reason (e.g. recent bereavement, terminal illness or unable to give informed consent).

### Selection of participants

Lists of eligible persons will be generated from practice registers. These lists will be scrutinised by GPs, who will remove all patients deemed inappropriate to receive a questionnaire (according to criteria defined above).

A postal questionnaire will assess symptoms over the past three months, the acceptability of screening tests for colorectal cancer, risk factors for colorectal cancer, and willingness to take part in the study. The symptoms are based on the Department of Health (DoH) referral guidelines for suspected colorectal cancer [23,24]. One reminder will be sent two weeks later. Respondents who describe any colorectal symptoms (except only abdominal bloating and/or anal symptoms) and express willingness to take part in the evaluation will be sent an information leaflet and an invitation to attend a research clinic at their general practice.

GPs will be notified by fax of people who report symptoms that meet the criteria for urgent referral under the two week standard [24]. Such people will continue to be eligible for the study, although the GP will be made aware of their symptoms before the person is invited to a general practice based research clinic. Regular reports of symptoms (except abdominal bloating and anal symptoms) reported by all respondents will be provided to GPs. Reports of all people meeting the criteria for urgent referral and who decide not to fully participate in the study will be regularly provided to their GP.

### Informed consent

At the general practice clinic, the study will be fully described including potential benefits and risks, fitness for colonoscopy will be checked, and a basic medical history will be obtained. Informed consent will be sought and an appointment for colonoscopy will be arranged for those providing consent.

### Clinical evaluations, laboratory tests and follow-up

Colonoscopies will be performed in a dedicated research endoscopy facility based in the Wellcome Clinical Research Facility. Participants will be informed of the colonoscopy result on the day and the result will also be sent to their GP. Any participant found to have a malignancy will receive usual care. Two 5 ml blood samples will be collected immediately before the colonoscopy. One sample will be sent to the haematology laboratory for routine analysis (Hb, etc.), the other sample is for MMP-9 determination During transport this samples will be stored at 4°C. Serum will be stored at -80°C and the MMP-9 level will be determined on duplicate aliquots of each sample by ELISA assay. Participants will be flagged on the NHS Central Register to maximise the ascertainment of malignancy in those with no abnormality detected on colonoscopy.

### Procedures for collecting and handling data

All data will be entered into a password-protected Access database and each participant will be allocated a unique study number. Access to the password-protected database will be restricted to core University of Birmingham staff working on this study, who are trained in policies and procedures related to confidentiality. The University is registered under the data protection act and university policies and procedures related to confidentiality will be followed.

Demographic details will be provided by the GP for eligible people aged 50–69 years. Data on symptoms, risk factors and the acceptability of screening tests for colorectal cancer will be collected using the recruitment questionnaire. Participants will also complete a bowel preparation acceptability questionnaire prior to colonoscopy and two post-test (colonoscopy) acceptability questionnaires (prior to discharge at 10 days subsequent to colonoscopy). Blood sample results and colonoscopy findings will be communicated to the study team using the unique study number and linked to patient records.

### Justification of sample size

All types of colonic polyps have potential for malignant change but this is more common with increasing size, a villous growth pattern and more severe dysplasia [25]. Increasing age, adenoma size and a villous component have been associated with risk for high grade dysplasia [26].

In the USA, 11% (n = 329) of 3121 people aged 50–75 years without symptoms of lower gastrointestinal disease who had a complete colonoscopy had advanced colonic neoplasia (adenoma with a diameter of at least 10 mm, or villous features, high grade dysplasia, or invasive cancer) [27]. If large tubular adenomas were excluded, about 6% (n = 155) of people had lesions. About 32% (n = 105) of the 329 people only had lesions proximal to the rectum and sigmoid colon.

In Norway, 13% (n = 25) of 193 people aged 62–73 years who were offered a colonoscopy or flexible sigmoidoscopy and had a full colonoscopy had a high risk adenoma (adenoma greater than or equal to 10 mm and/or villous components and/or severe dysplasia) [28]. About 39% (n = 11) of the 28 high risk lesions were not in the rectum or sigmoid colon. In the UK, high risk lesions (greater than or equal to three adenomas, a polyp greater than 1 cm, tubulovillous or villous histology, severe dysplasia, malignancy, or greater than or equal to 20 hyperplastic polyps) were found in 5% (n = 1905) of 40674 people aged 55–64 years who were screened using flexible sigmoidoscopy [9]. This will not include proximal lesions that could be found on colonoscopy.

Any test that might usefully be employed to screen for malignant or premalignant colorectal lesions should have a high sensitivity to minimise the false negative rate. Based on our pilot data and conservatively assuming a community prevalence of 6% [9,27,28]. a sample of 700 people would be sufficient to estimate sensitivity within 3% (95% confidence) and specificity within 4% (95% confidence). If the community prevalence of high risk lesions is greater than 6%, our estimate of sensitivity will have greater precision.

**Figure 1:**
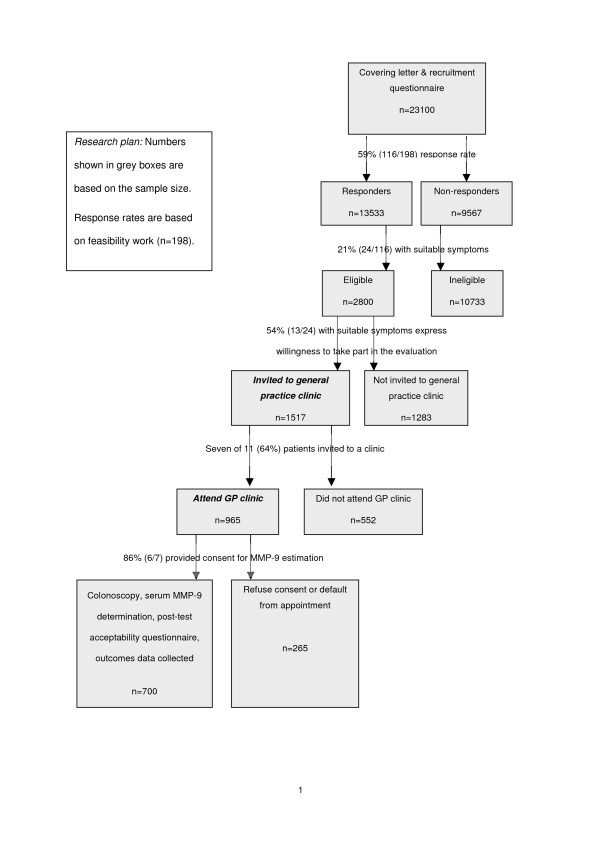
Research plan and study design.

Based on our pilot data (3.0% of the age group being suitable and consenting) and a practice list of 4500 with 18% of people aged 50–69 years [29]. 23100 people from 29 practices will be recruited to identify the 700 participants needed (Figure 1). The practices will be recruited via the Midlands GP Research Consortium (MidRec). This is a network of almost 400 research active practices with proven success in facilitating recruitment to primary care based trials. To maximise recruitment, the study has been designed to have minimal impact on general practices. Recruitment will be over 20 months.

### Methods of data analysis

#### Primary analysis

(1) ROC curve analysis will be undertaken to determine cut-off levels.

(2) Sensitivity, specificity, and positive and negative predictive values will be calculated with confidence intervals.

(3) The combination of MMP-9 level, symptoms, risk factors and socio-demographic status that best predict invasive and non-invasive colorectal neoplasia will be described.

(4) The acceptability of serum MMP-9 determination, FOB testing, flexible sigmoidoscopy and colonoscopy will be assessed by the initial questionnaire. The acceptability of serum MMP-9 determination and colonoscopy will also be assessed by a post-test acceptability questionnaire.

#### Secondary analysis

Logistic regression analyses of appropriate subgroups (for example age or symptom profile) will be used to identify where an alternative cut-off may be more appropriate.

### Bias and confounding

There will be duplicate determination of the serum MMP-9 level and dual data entry. Information on potential confounders, for example injuries or chronic illnesses that may lead to a raised serum MMP-9, will be collected at the general practice clinic. National and published data will be used to identify selection bias based on socio-demographic or symptom status. The analysis will be adjusted to take account of any confounders or selection bias. The blood sample for serum MMP-9 determination will be taken on the same day as the colonoscopy to improve comparability. All blood samples will be analysed in the same laboratory to ensure standardisation of measurement and reporting. The technician doing the MMP-9 ELISA assay and the clinician undertaking the colonoscopy will be blinded to the patient's symptoms as reported on the questionnaire.

### Ethical approval

This study has been approved by South Birmingham Research Ethics Committee: reference 05/Q2707/133.

## Discussion

Colorectal cancer is a major cause of morbidity and mortality. Most colorectal cancers arise from adenomas and there is a period for early detection by screening, but available tests have risks, are unacceptable to many, have high false positive rates, or are expensive.

A pilot study of patients attending a specialist clinic has indicated that serum MMP-9 is an accurate and acceptable test for colorectal cancer. The sensitivity and specificity of a test can, however, vary in populations with different disease spectrums. This study will evaluate the accuracy of serum MMP-9 as a test for colorectal cancer in a primary care population, the target population for screening.

This study will establish the potential of serum MMP-9 as a screening test for colorectal cancer. If it is confirmed as accurate and acceptable, this serum marker has the potential to assist with reducing morbidity and mortality from colorectal cancer.

## Competing interests

The author(s) declare that they have no competing interests.

## Authors' contributions

SW, MW, RH, AR, JD, AM and TI contributed to the original design of the study. AR drafted the study protocol with input from all authors. SW and JD provided statistical advice re justification of sample size and identified methods of data analysis to be utilised. SW drafted the manuscript. All authors read and approved the final manuscript.

## Pre-publication history

The pre-publication history for this paper can be accessed here:
